# Supplementary material for: Micro-mechanical fingerprints of the rat bladder change in actinic cystitis and tumor presence
Source: Commun Biol. 2023 Feb 24;6:217. doi: 10.1038/s42003-023-04572-0 (PMC9950451; doi:10.1038/s42003-023-04572-0)
Supplement: Supplementary file 2 — Supplementary Information [file 42003_2023_4572_MOESM2_ESM.pdf]

SUPPLEMENTARY INFORMATION for the manuscript "Micro-mechanical fingerprints of the rat bladder change in actinic cystitis and tumor presence"

Laura Martinez-Vidal, M. Chighizola, M. Berardi, E. Alchera, I. Locatelli, F. Pederzoli, C. Venegoni, R. Lucianò, P. Milani, K. Bielawski, A. Salonia, A. Podestà, and M. Alfano.

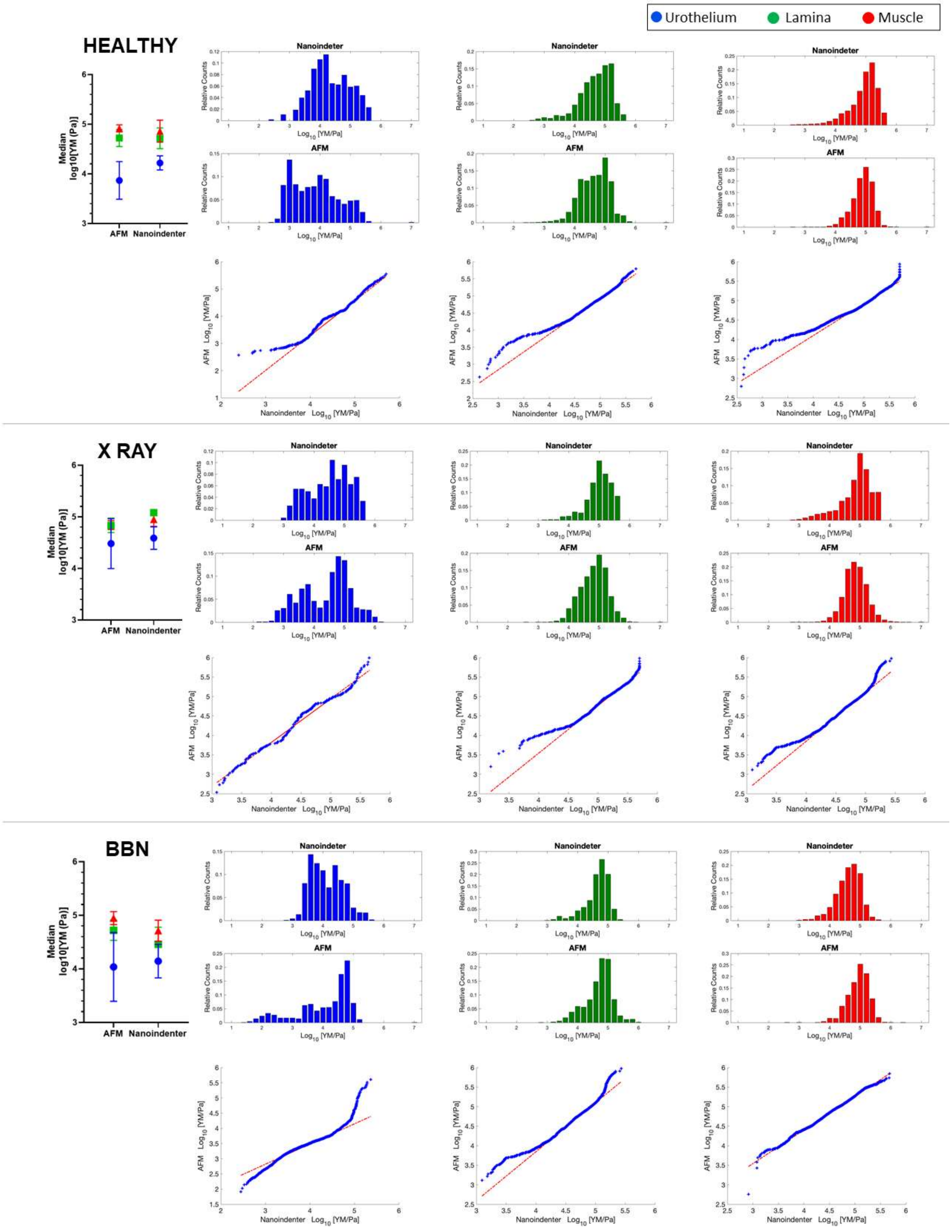

Supplementary Figure 1. Representation of YM data extracted by the two instruments (AFM and nanoindenter) of each single tissue layer, at month 2, extracted from healthy, X-ray irradiated and BBN rats, shown as: mean of the median values, distribution of single YM values, and quantile-quantile (QQ) plots comparing the two distribution

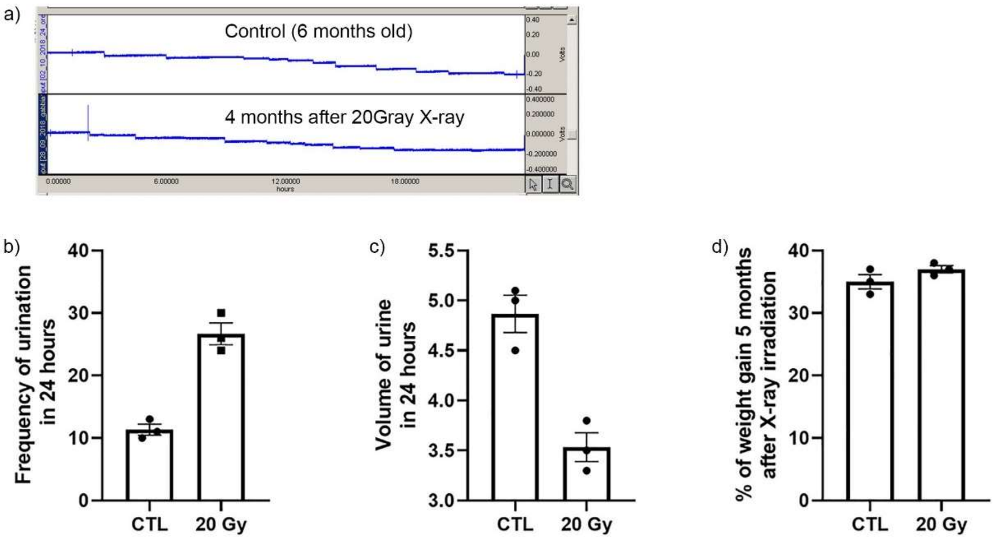

Supplementary Figure 2. Micturition in X-ray irradiated rats. A) Frequency and urine volume were monitored for 24 hours using metabolic cages connected with AC/DC strain gage amplifier (model CP122, GRASS Instrument Division, Astro-Med Inc., West Warwick US); one representative animal each condition is shown. Frequency of urination (b), urine volume (c) and percentage of weight gain (d) are shown for 3 animals each condition.

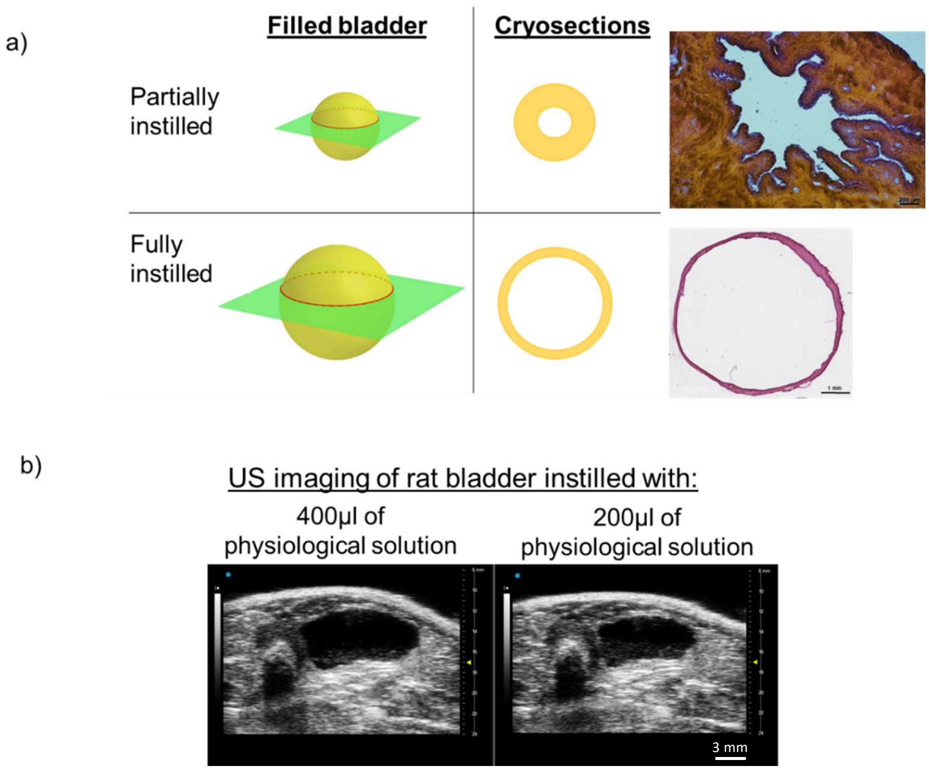

Supplementary Figure 3. Ultrasound imaging (US) for physiological volume determination. a) Scheme of partially and fully instilled bladders (left), photo of hematoxylin-eosin stained cryosections (right). b) US imaging of rat bladders instilled with different PBS volumes

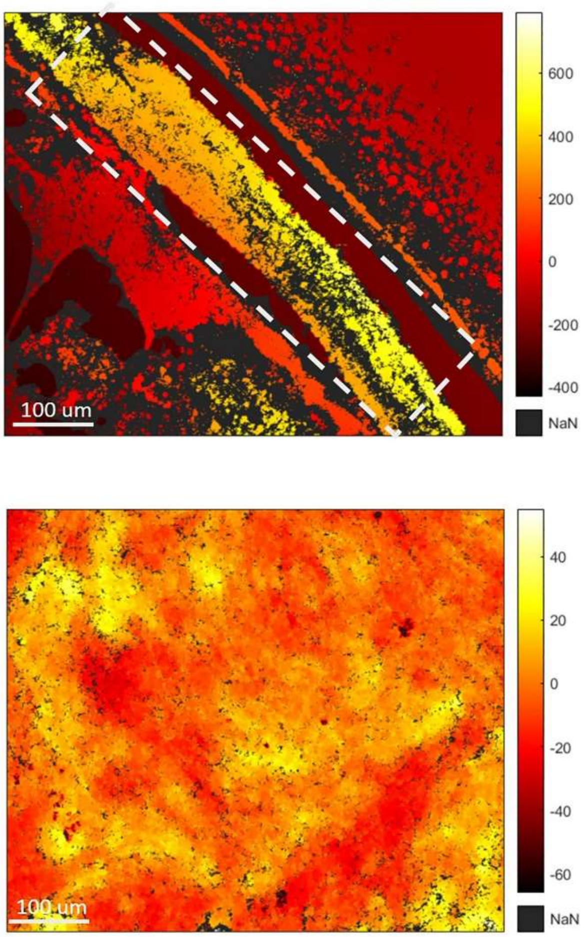

Supplementary Figure 4. Surface roughness image of bladder tissue cryosections. In order to ensure conformal contact between probe and sample, it is important to assess the surface roughness of the tissue under study. To quantify this, we characterized the topography of both rat (top) and human (bottom) samples using a non-contact approach. The images represent 620x480 um scans of the surface of frozen tissue samples. These were obtained with an optical scanning profiler, using white light illumination (Veeco Wyko NT9100). In the rat image, it is possible to see the arched bladder wall (brighter area highlighted by white dashed line) surrounded by the OCT. Heatmap represents elevation, in nm. In both cases, we found that the surface roughness was in the order of hundreds of nm (Ra ~200-400 nm) when analyzing small areas, approximately equal to the contact area during indentation, and in the order of microns in regions separated by hundreds of µm.

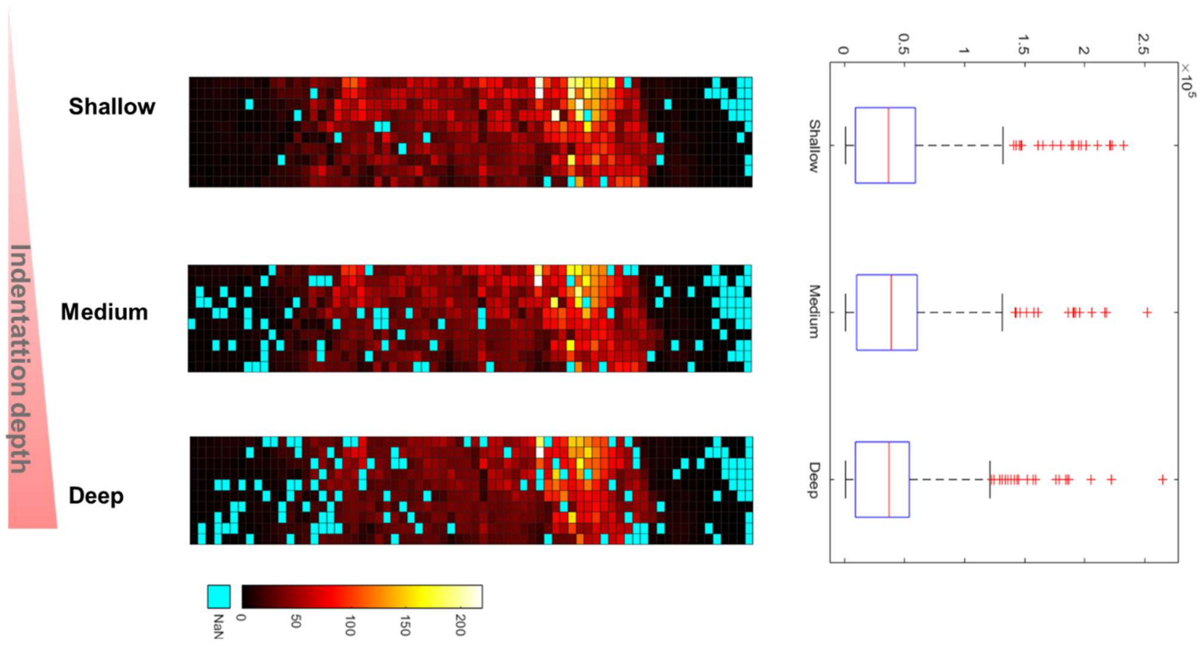

Supplementary Figure 5. YM's at different indentation depths obtained using the nanoindenter. YM's was analyzed at shallow (1.5 µm), medium (2.5 µm) and deep (5 µm) indentation depths. With increasing indentation depth, the number of non-valid pixels (blue) increased due to bottom effect, while YM values remain equivalent.
