# Supplementary material for: Micro-mechanical fingerprints of the rat bladder change in actinic cystitis and tumor presence
Source: Commun Biol. 2023 Feb 24;6:217. doi: 10.1038/s42003-023-04572-0 (PMC9950451; doi:10.1038/s42003-023-04572-0)
Supplement: Supplementary file 3 — Description of Additional Supplementary Files [file 42003_2023_4572_MOESM3_ESM.pdf]

Description of Additional Supplementary Files

File name: Supplementary Data 1

Description: the source data behind the YM's graphs in the paper. Excel file with legend of filename attached

File name: Supplementary Data 2

Description: the source data behind a representative control YM heatmap

File name: Supplementary Data 3

Description: the source data behind a representative X-ray irradiated bladder YM heatmap

File name: Supplementary Data 4

Description: the source data behind a representative bladder not responding to X-ray YM heatmap

File name: Supplementary Data 5

Description: the source data behind a representative Dysplastic bladder YM heatmap

File name: Supplementary Data 6

Description: the source data behind a representative pTa tumor YM heatmap

File name: Supplementary Data 7

Description: the source data behind a representative pT1 tumor YM heatmap

File name: Supplementary Data 8

Description: the source data behind the human patient histogram in the paper

File name: Supplementary Data 9

Description: the source data behind the collagen quantification graphs in the paper
